# HIV induces expression of complement component C3 in astrocytes by NF-κB-dependent activation of interleukin-6 synthesis

**DOI:** 10.1186/s12974-017-0794-9

**Published:** 2017-01-26

**Authors:** Jadwiga Nitkiewicz, Alejandra Borjabad, Susan Morgello, Jacinta Murray, Wei Chao, Luni Emdad, Paul B. Fisher, Mary Jane Potash, David J. Volsky

**Affiliations:** 1 Department of Medicine, Division of Infectious Diseases, Icahn School of Medicine at Mount Sinai, New York, 10029 NY USA; 2 Manhattan HIV Brain Bank, Department of Neurology, Icahn School of Medicine at Mount Sinai, New York, 10029 NY USA; 3 Department of Human and Molecular Genetics, VCU Massey Cancer Center, School of Medicine, VCU Institute of Molecular Medicine, Virginia Commonwealth UniversitySchool of Medicine, Richmond, 23298 VA USA; 4 Present Address: PSI-CRO, Wisniowy Business Park C, 1 Sierpnia 6A, 02-134 Warsaw, Poland; 5 Department of Medicine, Division of Infectious Diseases, 1468 Madison Avenue, Annenberg Building, 21st Floor, Room 42, New York, 10029 NY USA

**Keywords:** HIV, HAND, Complement component C3, Astrocytes, IL-6, Neurodegeneration, NF-κB, HIV-associated dementia, Brain tissue

## Abstract

**Background:**

Abnormal activation of the complement system contributes to some central nervous system diseases but the role of complement in HIV-associated neurocognitive disorder (HAND) is unclear.

**Methods:**

We used real-time PCR and immunohistochemistry to detect complement expression in postmortem brain tissue from HAND patients and controls. To further investigate the basis for viral induction of gene expression in the brain, we studied the effect of HIV on C3 expression by astrocytes, innate immune effector cells, and targets of HIV. Human fetal astrocytes (HFA) were infected with HIV in culture and cellular pathways and factors involved in signaling to C3 expression were elucidated using pharmacological pathway inhibitors, antisense RNA, promoter mutational analysis, and fluorescence microscopy.

**Results:**

We found significantly increased expression of complement components including C3 in brain tissues from patients with HAND and C3 was identified by immunocytochemistry in astrocytes and neurons. Exposure of HFA to HIV in culture-induced C3 promoter activity, mRNA expression, and protein production. Use of pharmacological inhibitors indicated that induction of C3 expression by HIV requires NF-κB and protein kinase signaling. The relevance of NF-κB regulation to C3 induction was confirmed through detection of NF-κB translocation into nuclei and inhibition through overexpression of the physiological NF-κB inhibitor, I-κBα. C3 promoter mutation analysis revealed that the NF-κB and SP binding sites are dispensable for the induction by HIV, while the proximal IL-1β/IL-6 responsive element is essential. HIV-treated HFA secreted IL-6, exogenous IL-6 activated the C3 promoter, and anti-IL-6 antibodies blocked HIV activation of the C3 promoter. The activation of IL-6 transcription by HIV was dependent upon an NF-κB element within the IL-6 promoter.

**Conclusions:**

These results suggest that HIV activates C3 expression in primary astrocytes indirectly, through NF-κB-dependent induction of IL-6, which in turn activates the C3 promoter. HIV induction of C3 and IL-6 in astrocytes may contribute to HIV-mediated inflammation in the brain and cognitive dysfunction.

**Electronic supplementary material:**

The online version of this article (doi:10.1186/s12974-017-0794-9) contains supplementary material, which is available to authorized users.

## Background

HIV-infected patients are at high risk of central nervous system diseases termed HIV-associated neurocognitive disorders (HAND), which include in a decreasing order of severity HIV dementia (HAD), mild neurocognitive disorder (MND), and asymptomatic neurocognitive impairment (ANI) [1]. HAD is a severe neurodegenerative brain disease which is often accompanied by the pathological manifestation of encephalitis (HIVE) and which presents with cognitive, motor, and behavioral symptoms [2]. ANI and MND are milder, chronic cognitive dysfunctions that generally do not progress to dementia [3] and are diagnosed solely by the extent of neurocognitive impairment (NCI) in neuropsychological tests [1, 4]. Wide access to antiretroviral therapies (ART) diminished the prevalence of HAD but had little effect on the milder forms of NCI which are now the predominant HIV brain dysfunctions seen in about 50% of patients on ART [5–8].

The HIV role in HAND is subject of intense research. Although HIV generally does not target neurons [9], the virus was shown to cause neuropathogenesis through production of neurotoxic viral proteins and cellular mediators secreted by HIV-infected cells, primarily macrophages, microglia, and astrocytes (reviewed in [10]). Some potential mediators of neuropathogenesis, including CCL-2, IL-8, and IL-6, can also be produced by astrocytes exposed to HIV, the HIV proteins Tat and gp120, or SIV [11–14]. Because astrocytes are the most numerous cells in the brain [15], their capacity to amplify neuropathogenic effects of HIV-infected macrophages and microglia in this manner may be significant.

In our investigation of activation of primary human fetal astrocytes (HFA) by HIV, we found that HIV binding is sufficient to induce transcription and secretion of IL-6 and IL-8 [16]. IL-6 is one of the cytokines elevated in the brain of individuals with HAND and it is considered a predictive marker of neuropathogenesis of SIV-infected macaques [17, 18] and in HIV-infected humans [19]. IL-6 may also be involved in synaptic function and brain pathologies [20–22]. To identify other potential pathogenic products of astrocytes, we investigated the global responses of these cells to HIV exposure [22] and to productive infection in culture by gene expression profiling [23]. Among the most highly induced cellular transcripts in infected HFA is complement component C3, the pivotal protein in the classical complement cascade, and other complement components. Precedents exist for the synthesis of C3 by astrocytes after HIV activation. Speth and colleagues reported induction of C3 by exposure of astrocytic cell lines to HIV as well as some of its proteins and investigated the mechanism of its induction [24–26].

Complement has been described as an important factor in the pathogenesis of many central nervous system diseases including infectious, autoimmune and degenerative disorders, and lately has been implicated in neuropsychiatric diseases [27–29]. Complement overexpression is associated with acute brain injury and chronic neurodegenerative diseases including Alzheimer’s disease [30–32] and Huntington’s disease [33]. Furthermore, C3 serves as a stage-biomarker of Alzheimer’s disease in CSF [34]. Elevated complement expression was also found in the brains of patients with HIV dementia [35], cerebrospinal fluid of patients with HAND [36–38], in the brain tissues of macaques with SIV encephalitis [39], and in the brain of mice infected with chimeric HIV [40], indicating that induction of complement may be part of the neuroinflammatory insult associated with HIV and SIV neuropathogenesis.

The role of elevated complement in HAND is unknown. One mechanism through which C3 may impair neuronal function has been suggested by studies showing that C1q and C3 are required for synaptic remodeling during brain development [41]. The authors suggested that aberrant expression of complement components in the adult brain might mediate inappropriate synaptic elimination, impairing neuronal function [41]. Consistent with this view, it is noteworthy that SIV-infected macaques display C3 deposition on neuronal membranes in the brain [42] and that antiretroviral treatment of macaques reduces the SIV-associated expression of complement components in the brain [43]. Recent studies in this regard suggest that the disruption of microglia CR3/C3 signaling results in sustained deficits in synaptic connectivity [44] and that complement and microglia mediate synaptic pruning and remodeling of synaptic connectivity in the brain [31, 45]. The synaptic loss mediated by complement and microglia was described in a model of frontotemporal dementia [46] and in a model of Alzheimer’s disease [47]. It also was associated to synaptic changes in multiple sclerosis [48] and schizophrenia [29, 49]. Vasek and colleagues described a similar process during synaptic loss in memory impairment after virus infection. Infection with West Nile virus induces complement-mediated elimination of presynaptic terminals [50]. The participation of astrocytes in this pathogenic process is indicated by findings that C3 secreted from astrocytes interacts with microglial C3a receptor (C3aR) to alter cognitive function in Alzheimer’s disease [51]. These findings suggest that complement is an important mechanism in synaptic remodeling in neuropathology and that astrocytes may play an important role in the process.

In this study, we investigated the physiological significance and mechanism of C3 overexpression by primary human astrocytes in response to HIV. The present work demonstrates elevated expression of complement components in the HIV-infected brain and delineates the cellular pathway through which activation occurs. We find that the initial response of primary astrocytes to HIV involves multiple protein kinases and NF-κB-dependent induction of IL-6; in turn, IL-6 appears to promulgate the response by activating expression of C3. Intervening at pivotal sites in this network can interrupt the spread of pathogenic responses from cell-to-cell and reduce HIV neuropathogenesis.

## Methods

### Patients and brain samples

Adult human brain specimens were provided by the Manhattan HIV Brain Bank, a member of the National NeuroAIDS Tissue Consortium (U24MH100931) under an Institutional Review Board-approved protocol at Icahn School of Medicine at Mount Sinai. HIV and gene expression analyses were conducted on coded brain samples without subject identifiers under an “exempt” status approved by an Institutional Review Board of St. Luke’s-Roosevelt Hospital Center (presently Mount Sinai). Samples from four HIV-positive and three HIV-negative subjects were used in this study. All HIV-positive patients had evidence of HIVE; HIV-negative subjects (controls) had normal neurological function and unremarkable brain histology. Ages ranged from 30 to 63 years; five were male and two were female; and three were white, two hispanic, and two black. All samples used for analyses were from a similar location within gray matter of the frontal lobe.

### Analysis of brain samples

RNA was extracted using the RNeasy Mini Kit (QIAGEN, Valencia, CA). cDNA was prepared from individual brain samples using the WT-Ovation™ RNA Amplification System (NuGEN Technologies, Inc., San Carlos, CA). QPCR was conducted using TaqMan chemistry and using probes from the Universal Probe Library (Roche Diagnostics, Indianapolis, IN) and primers designed with the online ProbeFinder software (https://lifescience.roche.com/en_us/brands/universal-probe-library.html). In the PCR reaction, 5 μl of cDNA obtained as previously described was combined with 10 μl of 2X Universal Master Mix (Thermo Fisher Scientific, Waltham, MA), 0.2 μl of each forward and reverse primers at 200 nM, 0.2 μl of probe at 100 nM, and RNAse/DNAase-free water. All reactions were performed in duplicate and were run in a 7500 real-time PCR system (Thermo Fisher Scientific). Data were normalized using glyceraldehyde-3-phosphate dehydrogenase (GAPDH). For immunohistochemical analysis, formalin-fixed blocks were used to construct tissue microarrays with three punches (diameter of 1 mm) from each block. Five-micrometer sections were cut and immunohistochemistry performed using a rabbit polyclonal anti-C3c complement antibody (1:3000, DakoCytomation) and either mouse anti-GFAP or anti-CD68 as the primary reagents and diaminobenzidine or amino ethyl carbazole as the chromogens.

### Cell culture, HIV preparation, and infection

HFA were isolated from second trimester human fetal brains obtained from elective abortions in full compliance with NIH guidelines as previously described [52]. Highly enriched populations of astrocytes were obtained by high-density cultures condition in the absence of growth factors in F12 Dulbecco’s modified Eagle’s medium (Thermo Fisher Scientific), containing 10% fetal bovine serum (Hyclone, Piscataway, NJ) antibiotics and fungizone. Cells were incubated at 37 °C in 5% CO_2_/95% air. HIV-1 molecular clones used were NL4-3 (clone pNL4-3; M19921) from Dr. Malcolm Martin’s laboratory [53] that expresses all HIV-1 proteins. HIV/NL4-3 stocks were prepared by transfection of 293T cells. Cells were transfected by calcium phosphate co-precipitation method [54] and then purified by sedimentation as described; mock virus was sedimented in parallel from supernatants of 293T cells [52]. Residual plasmid DNA was removed post-transfection using DNAse I digestion (Sigma, St. Louis, MO). Early passages of HFA were cultured for 5–7 days until 75% confluence; the cells were washed in warm PBS and infected with cell-free HIV-1 at the indicated number of picograms per cell or comparable dilutions of mock virus for 2 h at 37 °C and were washed three times to remove the virus. For more detailed protocol, see [55] and [16].

### Detection and quantification of IL-6 or C3 by ELISA

Cell supernatants were collected as indicated and the levels of IL-6 were measured by ELISA (Raybiotech, Norcross, GA). To detect C3, a (indirect) sandwich ELISA was conducted using goat anti-human C3 polyclonal antibody (Sigma) as the antibody in solid phase, followed by cell supernatants. Bound C3 was detected using rabbit anti-human C3c complement antibody (Dako, Carpinteria, CA), as the detection antibody followed by peroxidase conjugated-monoclonal anti-rabbit IgG (γ-chain specific) (Sigma). The concentration of C3 protein was measured using complement C3 protein from human serum (Sigma) as a standard (in serial dilution).

### Luciferase reporter plasmids containing C3 promoter or mutant promoter constructs

The C3 promoter region was cloned by PCR amplification of human astrocyte DNA using primers: sense (5′ GCGCGCTAGCCTGCAATTTAGCCTGAGTGACAGAATG 3′) and antisense (5′ GCGCCTCGAGAGAGGGACAGAGGGACAGAGGGAGAGG 3′); the sense primer contains a *Nhe*I cleavage site and the antisense primer contains a *Xho*I cleavage site for insertion into the pGL3 vector (Promega, Madison, WI). The reaction was performed in a buffer containing 10 mM Tris-HCl, 50 mM KCl, 0.1% Triton X-100, 200 μM each dNTP, 2.5 mM MgCL_2_, 4 units Taq polymerase (Promega), 0.5 μg DNA, and 0.6 μM each primer, in total reaction of 50 μL. The cycling parameters were 94 °C for 1 min, 55 °C for 1 min, and 72 °C for 2 min, for a total 25 cycles for both stages. Six C3 promoter deletion mutants were constructed by PCR amplification using sense primers containing a *Nhe*I cleavage site as follows:

- C3P50: 5′ GCGCGCTAGCTGGGGGAAAGGCAGGAGCCAGATAA 3′
- C3P100: 5′ GCGCGCTAGCCTGGGGCAGCCCCAAAAGGGGAGAGG 3′
- C3P200: 5′ GCGCGCTAGCAGCTGCATTCATGCTGCTGGGGAAC 3′
- C3P300: 5′ GCGCGCTAGCCTCCAGACCTTAGTGTTCTTCCACTAC 3′
- C3P600: 5′ GCGCGCTAGCCAGGAAGTTTTCCCTGACCCTCCAAG 3′
- C3P875: 5′ GCGCGCTAGCGATCAATATGAATATATTATACACACAG 3′

and an antisense primer containing a *Xho*I cleavage site (5′ GCGCCTCGAGAGCAGCGCCTGCTGGAGCTGGCTTTTTATC 3′). Six replacement mutations were constructed in specific response elements within the C3 promoter by PCR. The table contains the mutagenic primers and sequences altered. Reaction products were inserted into the pGL3 basic vector.

### IL-6 promoter constructs

Dr. Gail Bishop of the University of Iowa (Iowa City, IA) kindly provided plasmids encoding luciferase whose expression is driven by the intact murine IL-6 promoter or a mutated promoter lacking the NF-κB recognition element [56]. Their function in HFA was assessed as described for constructs encoding C3 promoter-dependent luciferase expression.

**Table:** 

Mutants	Primer	Sequence	Position/response element
M1	5′ GGAAATGGTATTGGAGGATCTGGGGCAGCC 3′ 5′ GGCTGCCCCAGATCCTCCAATACCATTTCC 3′	ATTG**AGAA**ATCTGGGGCAG; ATTG**GAGG**ATCTGGGGCAG	−109–(−90); IL-1β/IL-6
M2	5′ GGAAATGGTATCAGGAAATCTGGGGCAGCC 3′ 5′ GGCTGCCCCAGATTTCCTGATACCATTTCC 3′	**TGA**GAAA; **CAG**GAAA	−106–(−100); IL-6
M3	5′ GAAAAGCTTAGGGGGTGGTATTGAGAAATC 3′ 5′ GATTTCTCAATACCACCCCCTAAGCTTTTC 3′	GAAA**TGGT**----ATTGAGAA; GAAA**AGCT**----ATTGAGAA	−116–(−100); IFN-γ
M4	5′ CATTCATGCTGCTAAAGAACATGCCCTCAG 3′ 5′ CTGAGGGCATGTTCTTTAGCAGCATGAATG 3′	T**GGG**GAA; T**AAA**GAA	−181–(−175); IL-6
M5	5′ CCCATCTGAAATGCTTCCTCCTACAGGAAG 3′ 5′ CTTCCTGGTAGGAGGAAGCATTTCAGATGGG 3′	GG**GGA**CATTTCA; GG **AAG**CATTCA	−615–(−604); NF-κB
M6	5′ CTCTAGAAATGAAAGCTTTCCTCAGTGATG 3′ 5′ CATCACTGAGGAAAGCTTTCATTTCTAGAG 3′	GA**GGA**C-TTTCC; GA**AAG**C-TTTCC	−750–(−740); NF-κB

### QPCR for detection of C3 gene expression in HFA, and other cellular gene expression in human brain tissue

RNA was isolated from astrocytes with RNeasy kits (QIAGEN), and cDNA was synthesized using the Superscript kit (Thermo Fisher Scientific), all according to manufacturer’s instructions. Primers for C3 and GAPDH were purchased from Applied Biosystems (Thermo Fisher Scientific). Primers for other human genes were designed using the Roche Universal Probe Library Assay Center and were purchased from Thermo Fisher Scientific; the Universal Probe Library (Roche) was used to provide probes. C3 gene expression in HFA was evaluated by fold change of Relative Quantification. In the human brain tissue study, QPCR reactions were prepared as described in [35]. All reactions were performed in duplicate. Raw data was analyzed using the 7900 System SDS Software (Thermo Fisher Scientific). Data was normalized using GAPDH expression values. Relative quantification employed the comparative threshold cycle method. Student’s *t* test was used to test significant *versus* control groups.

### Analysis of promoter function by luciferase activity

HFA were grown to 80% confluence in 12-well plates and transfected with plasmid DNA as follows: 1.5 μg C3 or IL-6 promoter driving firefly luciferase and 0.5 μg of *Renilla* luciferase vector, after 2.5 h of transfection using lipofectamine 2000 (Thermo Fisher Scientific), cells were washed and incubated 48 h with various stimuli, then lysed and both luciferase activities were measured using the Promega Dual Luciferase Assay kit according to the manufacturer’s instructions, firefly luciferase is reported as relative light units (RLU), normalized to *Renilla* luciferase activity.

### Inhibitors of signal transduction pathways

Astrocytes were preincubated for 6 h with one of pharmacological inhibitors (EMD Chemicals, Gibbstown, NJ) of signal transduction pathways or with vehicle as indicated: AG17 2 μg/ml AG18 10 μg/ml, CAPE 0.5 μg/ml, genistein 25 μg/ml, JNK inhibitor II 1 μg/ml, PDTC 5 μM, SB 202190 10 μM, SB 203580 10 μM, U0126 10 μM, and wortmannin 0.1 μg/ml. After preincubation with inhibitor, cells were washed and then were cultured in 7.5% FBS DMEM with/or without inhibitor, followed by HIV infection or mock control. Alternatively, HFA were infected with adenovirus control or an adenovirus expressing super-repressor I-κBmt32 as described [57]; cells were then transfected with the C3-luciferase construct, followed by HIV or mock infection and luciferase activity measured.

### Detection and quantification of NF-κB

For quantitation of nuclear content of NF-κB, nuclei were isolated using the Panomics Nuclear Extraction Kit and protein was measured using the Transbinding TM NF-κB Assay Kit according to the manufacturer’s instructions. Alternatively, astrocytes were cultured on two-well chamber slides, fixed with 4% formaldehyde, permeabilized with 0.1% Triton X-100 and after blocking nonspecific binding with 1% bovine serum albumin, stained with anti-p65 antibody (1:100; Santa Cruz Biotechnology, Santa Cruz, CA) overnight at 4 °C. Cells were then rinsed three times for 5 min each in PBS and incubated with Alexa488-conjugated anti-rabbit IgG (Thermo Fisher Scientific) for 1 h at room temperature. After three rinses for 5 min each in PBS, cells were mounted in Vectashield fluorescence mounting medium containing 4.6-diamidino-2-phenylindole (Vector Laboratories, Burlingame, CA). Images were taken with a Confocal Laser Scanning Microscope LSM Multiphoton 510 (Zeiss, Thornwood, NY).

### Statistics

**Fig. 1:**
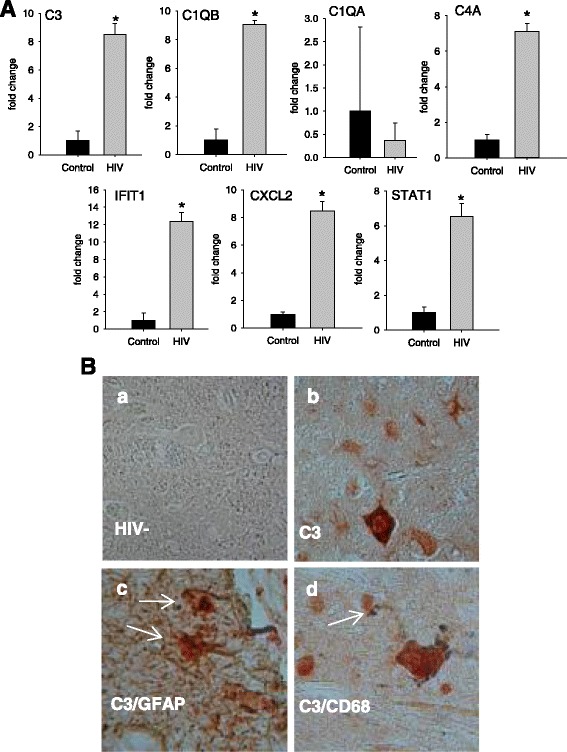
Upregulation of C3 and identification of producer cells in HIV-infected brain. **a** cDNA was prepared from the brain tissue from four patients who died with HIV dementia or three HIV-negative patients and subjected to QPCR amplifying the designated transcripts, data are expressed as fold change relative to uninfected tissue. The significance of gene induction of HIV-infected vs. uninfected was tested by Student’s *t* test with *asterisk* indicating *p* < 0.05. **b** Brain sections from an HIV-negative person (*a*) or an HIV-positive person (*b–d*) were stained for C3c expression (*b*) and either GFAP (*c*) or CD68 (*d*). The objective magnification used is ×40. See text for details

**Fig. 2:**
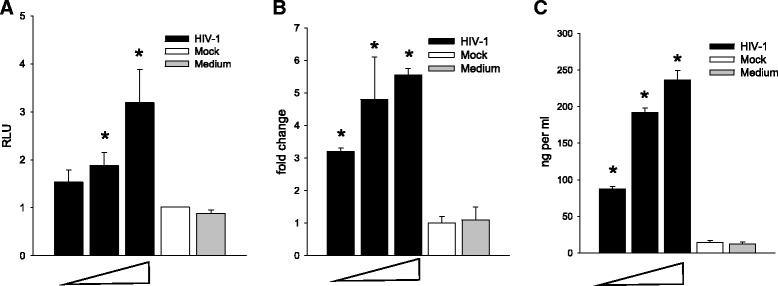
HIV activates the C3 promoter inducing transcription and protein production by HFA. HFA were exposed to NL4-3 at 0.5, 1.0, and 2.5 pg per cell or mock-infected and samples collected to measure C3 products. **a** Activation of C3 promoter directed luciferase production was measured 2 days after virus exposure; data are expressed as relative light units (RLU). **b** C3 mRNA was measured by QPCR 1 week after virus exposure. **c** Extracellular C3 protein was measured by ELISA 1 week after virus exposure. The differences in values between mock-infected and the other groups were tested by one-way ANOVA and Dunnett’s post hoc analysis with *asterisk* indicating *p* < 0.05. Statistical values are provided in Additional file 1: Table S1

**Fig. 3:**
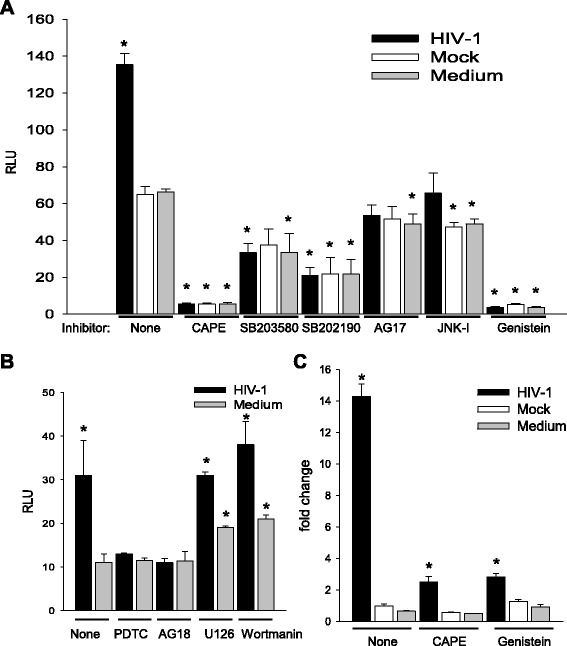
HIV induces multiple elements for activation of the C3 promoter in HFA. HFA were treated with pharmacological agents as indicated prior to exposure to medium, HIV, or mock virus and luciferase construction transfection. **a**, **b** Activity of the C3 promoter was assayed by luciferase expression, data are expressed as RLU. **c** Data are expressed as fold change relative to mock-infected cells. The differences in values between the untreated mock-infected (**a**, **c**) or untreated medium (**b**) and the other groups were tested by one-way ANOVA and Dunnett’s post hoc analysis with *asterisk* indicating *p* < 0.05. Statistical values are provided in Additional file 1: Table S1

**Fig. 4:**
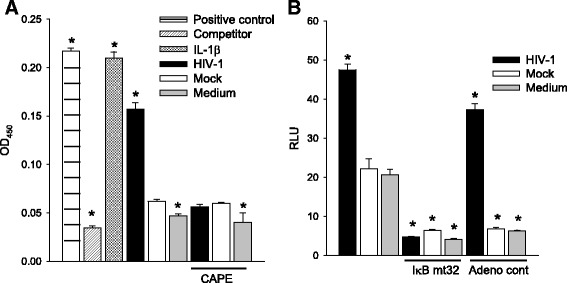
C3 induction by HIV in HFA requires activation of NF-κB. HFA were treated as indicated and **a** assayed for translocation of NF-κB into the nucleus by ELISA or **b** assayed for C3 promoter activity in the presence of adenovirus control vector or vector of dominant negative I-κBmt32. The differences in between untreated mock-infected and the other experimental groups were tested by one-way ANOVA and Dunnett’s post hoc analysis with *asterisk* indicating *p* < 0.05. Statistical values are provided in Additional file 1: Table S1

**Fig. 5:**
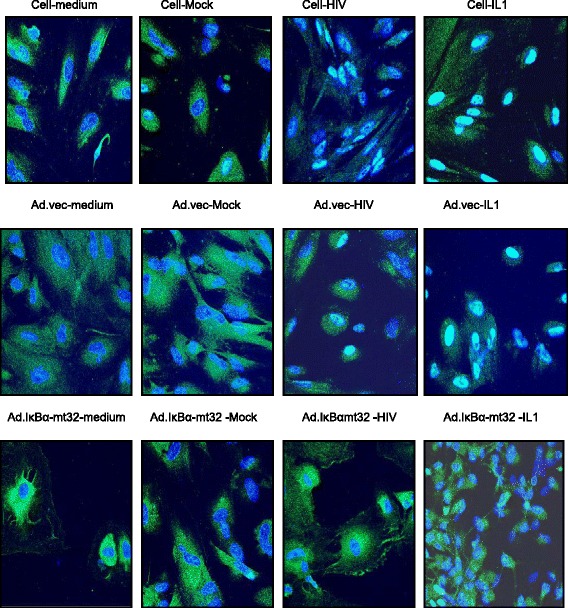
HIV induces nuclear translocation of NF-κB in HFA. HFA were treated as indicated and subjected to confocal microscopy, staining for DNA (*blue*) and the p65 chain of NF-κB (*green*). Please see the “Methods” section for details

**Fig. 6:**
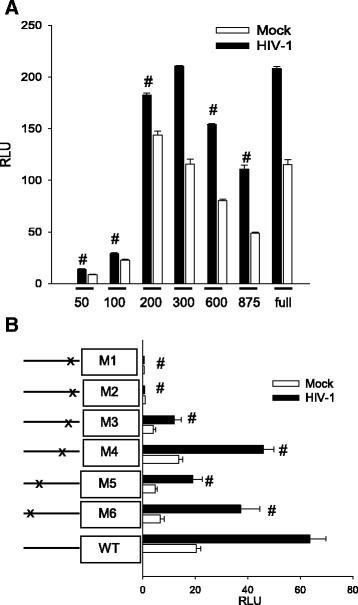
Elements in the C3 promoter essential for activation of HFA by HIV. HFA were exposed to HIV or mock virus and then transfected with various mutant C3 promoters driving luciferase production. **a** Truncation mutants in the C3 promoter. **b** Point mutants in the C3 promoter. The differences in values between the intact C3 promoter and deletion or point mutations (*HIV-1*) were tested by one-way ANOVA and Dunnett’s post hoc analysis with *number sign* indicating *p* < 0.05. Statistical values are provided in Additional file 1: Table S1

**Fig. 7:**
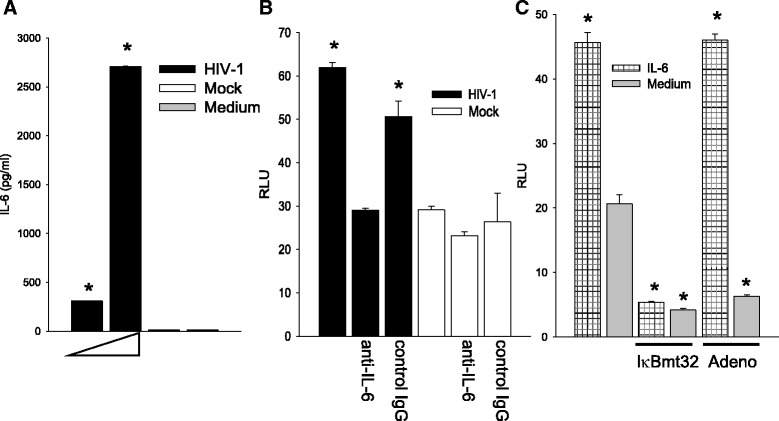
The induction of IL-6 is essential for the induction of C3 by HIV in HFA. **a** HFA were exposed to HIV at 0.5 and 2.5 pg per cell or mock virus and extracellular IL-6 was measured by ELISA. **b** HFA was exposed to HIV in the presence of anti-IL-6 or control antibody; cells were then transfected with the C3 promoter luciferase construct and luciferase activity was measured 2 days later. **c** HFA were infected with adenoviruses, treated with IL-6, and then transfected with C3 promoter constructs to score promoter activity by luciferase expression. The differences in values between mock-infected and HIV-infected cells or between IL-6 and medium were tested by one-way ANOVA and Dunnett’s post hoc analysis with *asterisk* indicating *p* < 0.05. Statistical values are provided in Additional file 1: Table S1

**Fig. 8:**
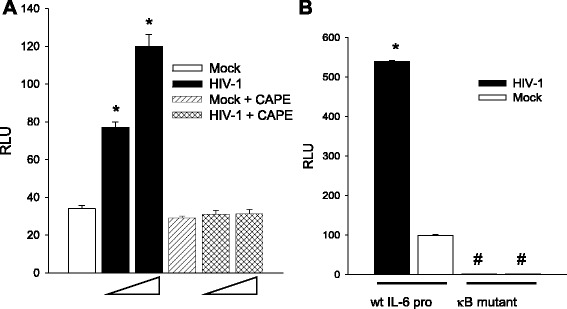
HIV activates the IL-6 promoter through NF-κB. **a** HFA transfected with the IL-6 promoter driving luciferase were exposed to HIV at 0.5 or 2.5 pg p24 per cell in the presence or absence of CAPE. Luciferase activity was measured and the difference in values between mock-infected and the other experimental groups **a**. Luciferase activity was measured for HFA transfected with the IL-6 promoter driving luciferase were exposed to HIV at 0.5 or 2.5 pg per cell or mock virus in the presence or absence of CAPE **a** and IL-6 promoter and a mutant in the κB site **b**. The differences in values between mock-infected and HIV-infected cells were tested by **a** one-way ANOVA and Dunnett’s post hoc analysis with *asterisk* indicating *p* < 0.05 and by Student’s *t* test with *asterisk* indicating *p* < 0.05 and **b** *number sign* for NF-κB mutant vs. wild type. Statistical values are provided in the Additional file 1: Table S1

Student’s *t* test was used to test significant differences in between two groups with asterisk indicating *p* < 0.05 (Figs. 1 and 8b). For all other studies (Figs. 2, 3, 4, 5, 6, 7, and 8a), one-way analysis of variance (ANOVA) was used to assess significant differences among the groups. Dunnett’s multiple comparison post hoc test was performed generally to compare all the groups to untreated mock-infected or untreated medium or in Fig. 6 to compare differences in values between the intact C3 promoter and deletion or point mutations in HIV infected. Significance is indicated by asterisk (*p* < 0.05). All the ANOVA and Dunnett’s statistical comparisons and values are included in Additional file 1: Table S1.

## Results

### Expression of C3 protein in HFA and in acute phase reactants in the brain from HIV-infected patients

Our results and observations by other investigators indicate that HIV exposure of transformed or primary human astrocytes in culture leads to induction of C3 [23–26]. To address the physiological significance of these findings in HIV neuropathogenesis, we measured the relative levels of the transcripts of C1qa, C1qb, C3, and C4a in brain tissue obtained at autopsy from individual patients with HIVE in comparison to brain tissue from uninfected individuals (Fig. 1A). C3,﻿ C1qb, and C4a transcripts, but not C1qa, were significantly upregulated in the brains of HIVE patients compared to controls. Other markers of inflammation, some previously implicated in HAD [23, 35, 58, 59], were also transcriptionally induced in the brains of HIVE patients including interferon-related genes IFIT1 and STAT1 and the *macrophage inflammatory protein 2-alpha* (MIP2-α), also known as the chemokine CXCL2.

To identify the cell types producing C3, we performed two-label immunohistochemistry staining on paraffin sections from control and HIVE brains for C3c and either GFAP for astrocytes or CD68 for microglia/macrophages (Fig. 1B). C3c was detected with AEC-conjugated antibody (red) and GFAP and CD68 with DAB (brown/black). Brain sections from an HIV-negative subject showed no C3c immunoreactivity indicating no baseline complement activation (Fig. 1Ba). In contrast, brain sections from a patient with HIVE (Fig. 1Bb–d) showed strong C3c staining, particularly in neurons (dark red cells in Fig. 1Bb and Bd) and some astrocytes (light red). Double staining for C3 and GFAP (Fig. 1Bc) confirmed the astrocytic expression of C3 during HIV infection in patients (red C3 staining in cell body and brown GFAP staining in cell processes; arrows). There was limited cellular co-localization of C3c and CD68 markers (Fig. 1Bd; arrow) suggesting that in this tissue, macrophages/microglia were not the major source of C3. These findings indicate that the environment of the HIV-infected brain generates signals to neurons and astrocytes to produce elevated C3. The remainder of this study is dedicated to identifying the specific triggers to this innate immune response.

### HIV activates the C3 locus in primary astrocytes in an NF-κB-dependent pathway

We established a primary tissue culture system to assay the responses of HFA to HIV/NL4-3 exposure at multiple levels of C3 regulation: protein, RNA, and promoter activation (Fig. 2). Cells were exposed either to mock virus concentrates as negative controls or medium for baseline expression. C3 promoter activity was measured by activation of the C3 promoter-driven luciferase marker (Fig. 2a); C3 RNA was measured by QPCR, standardizing by GAPDH levels (Fig. 2b); and extracellular C3 protein was measured by ELISA (Fig. 2c). At each level studied, HIV-induced C3 in HFA: HIV activated the C3 promoter, C3 transcription, and C3 protein expression.

As a first step to understand the mechanism of C3 activation, we began investigation of the protein kinase signaling pathways in HFA required for C3 induction. HFA were pretreated with pharmacological inhibitors of specific protein kinases, then were infected by HIV/NL4-3, and transfected with the C3 promoter-luciferase construct to assay the activity of the C3 promoter (Fig. 3). NF-κB inhibitors (CAPE and PDTC) as well as inhibitors of p38 (SB203580, SB202190), protein tyrosine kinase (Genistein), JNK, platelet-derived growth factor receptor tyrosine kinase (AG17), and epidermal growth factor receptor tyrosine kinase (AG18) blocked the HIV activation of transcription from the C3 promoter. Based upon the maintenance of activation of the C3 promoter in the presence of specific inhibitors, PI3K and MEK are not involved in induction of C3 by HIV in astrocytes. Earlier studies using astrocytic cell lines implicated cyclic AMP, protein kinase C, and C/EBPδ but not NF-κB in the activation of C3 expression in astrocytes by HIV [24]. To supplement the evidence obtained using pharmacological inhibitors (Fig. 3), we performed three complementary assays to investigate the state of activation of NF-κB after exposure of HFA to HIV. First, nuclear localization of p50 monomer of NF-κB in astrocytes was measured by ELISA after various stimuli (Fig. 4a). Like the positive control IL-1β, HIV induced the entry of NF-κB into the nucleus and this event was inhibited by CAPE. Second, we employed an adenovirus vector of super-repressor I-κBmt32 with the expectation that the complex of NF-κB and I-κBmt32 will resist degradation and NF-κB will be retained in the cytoplasm and cannot function in transcription. HFA were infected by control adenovirus or adenovirus encoding I-κBmt32 and then infected with HIV and induction of the C3 promoter was measured by luciferase assay (Fig. 4b). Like the experiment shown in Fig. 2, HIV exposure activated transcription from the C3 promoter; the control adenovirus vector had no effect upon this activation. In contrast, expression of I-κBmt32 and inhibition of activation of NF-κB completely blocked C3 promoter induction by HIV in HFA (Fig. 4b). Finally, to unambiguously demonstrate that HIV activates NF-κB in astrocytes, we localized NF-κB in HFA by confocal microscopy. Cells were activated by various stimuli in the presence of control adenovirus or adenovirus encoding I-κBmt32 super-repressor of NF-κB. Cells were fixed and stained for the p65 chain of NF-κB, nuclei were visualized with DAPI (Fig. 5). In the absence of stimuli, NF-κB is mainly cytoplasmic in HFA; however, both HIV and IL-1β induced the translocation of p65 into the nucleus (upper row). In contrast, the expression of the super-repressor I-κBmt32 arrested NF-κB nuclear localization induced either by HIV or by IL-1β (lower row); control adenovirus had no effect (middle row). The findings in Figs. 3, 4, and 5 demonstrate unequivocally that HFA respond to HIV by NF-κB-linked C3 synthesis. Further studies dissect the elements in the C3 promoter that control HIV-induced C3 responses.

We prepared a series of deletion and point mutations in the C3 promoter in the luciferase construct and tested their response during activation of HFA by HIV (Fig. 6). The minimal promoters consisting of only 50 or 100 base pairs upstream of the initiation codon were inactive; restoring the promoter to 200 base pairs restored the response to HIV (Fig. 6a). The constitutive activity of this construct was somewhat higher than that of the intact promoter, suggesting that there is a negative regulatory element upstream. To identify essential sites within the promoter recognizing transcriptional modulators, we prepared five mutations (Fig. 6b). Mutation of the proximal IL-1β/IL-6 site (M1 or M2) abolished both constitutive and HIV-induced transcription directed by the C3 promoter. In contrast, mutation of the interferon-γ response element (M3), the more distal IL-6 element (M4), or either of two NF-κB sites (M5 or M6) had only modest effects upon the C3 promoter response to HIV by HFA. Studies in the previous section demonstrated that HIV-induced C3 through NF-κB, but here it is clear that the NF-κB sites in the C3 promoter are dispensable for the response to HIV. This apparent paradox can be resolved if NF-κB is required for induction of expression of an intermediate protein that itself induces C3.

We propose that HIV activates IL-1β or IL-6 expression through NF-κB and one or both of these cytokines then initiates a program to activate the C3 promoter in HFA. As shown in Fig. 1, both IL-1β and IL-6 are prominently expressed in the HIV-infected brain, consistent with the premise that these cytokines trigger C3 production by astrocytes. We have previously shown that HIV can induce IL-6 transcription and secretion [16] and Fig. 7a illustrates dose-dependent induction of IL-6 in HFA by HIV. Because HFA do not produce IL-1β under the same conditions (not shown), we confined further studies to activities of IL-6 upon HFA. To definitively link the production of IL-6 to the activation of C3 expression, we employed anti-IL-6 neutralizing antibody. HFA expressing the C3 promoter-luciferase construct were exposed to HIV in the presence of anti-IL-6 or control antibody (Fig. 7b). The ability of HIV to activate the C3 promoter was reduced by neutralization of IL-6 to background levels, indicating that IL-6 is an essential intermediate in C3 induction by HIV. To determine whether IL-6 employs NF-κB to activate the C3 promoter, we exposed cells to exogenous IL-6 and inhibited NF-κB through transduction of the super-repressor, I-κBmt32 (Fig. 7c). IL-6 increased the activity of the C3 promoter in HFA but infection with the adenovirus vector of I-κBmt32 entirely blunted this response; infection with control adenovirus had no effect upon the C3 promoter response. These findings strongly implicate endogenously produced IL-6 in the pathway of HIV activation of C3 by HFA. To determine the transcriptional pathway employed in HIV activation of IL-6 synthesis in HFA, we tested the induction of the IL-6 promoter by pharmacological inhibition of NF-κB or by mutagenesis. An IL-6 promoter construct directing synthesis of luciferase was tested for activation by HIV in the presence or absence of CAPE (Fig. 8a) or an intact IL-6 promoter, and a mutant in the κB site driving luciferase expression were used (Fig. 8b). HIV required NF-κB activity to activate the IL-6 promoter, the presence of CAPE or a mutated κB element abolished promoter function. These findings, coupled with those in Fig. 7, strongly suggest that HIV activates a circuit in astrocytes, first inducing NF-κB-dependent IL-6 synthesis; extracellular IL-6 then initiates a secondary signaling cascade resulting in C3 transcription and protein expression.

## Discussion

The major finding of this work is that HIV initiates an auto-stimulatory pathway in cultured HFA in which NF-κB is activated to induce IL-6 production and IL-6 further signals to induce C3 transcription and protein production. IL-6 and C3 are also elevated in the brains of patients with HAND; double staining indicated that C3 was expressed by astrocytes. In culture, these responses involve multiple protein kinases and NF-κB, which are characteristic of the complexity of induction of other acute phase reactants, like serum amyloid A [60].

Several approaches have previously shown that HIV and SIV neuropathogenesis correlates with elevated expression of complement proteins in the central nervous system [32, 35, 36, 39, 61]. Immunohistochemistry staining in SIV-infected macaque brain tissues localized elevated expression of complement component proteins C3 and C1q to astrocytes, neurons, and myeloid cells [42]. We show by immunostaining here that complement C3c can also be overexpressed in astrocytes and cells with neuronal morphology in the brain tissues from patients with HAD/HIVE; these tissues also had elevated levels of C3, C4a, but not C1qa transcripts and, consistent with other studies [62], increased expression of inflammatory effector genes IL-1β and IL-6 (Fig. 1). At present, there is less information about potentially aberrant complement expression in the CNS of patients with mild HIV cognitive disease [63]. However, CSF and brain analyses from these patients revealed markers of increased neuroinflammation [64, 65] and complement activation is part of the inflammatory response to HIV infection [63]. Recent research also shows that HIV persists in astrocytes during presymptomatic stage of HIV infection [66], supporting the notion that HIV infection of these cells and potentially complement activation by the virus may impact the course of HIV neuropathogenesis.

Consistent with previous studies in astrocytic cell lines [24, 26], exposure of HFA to HIV in culture activates C3 promoter in a virus dose-dependent manner resulting in increased C3 mRNA and production of secretable C3 protein (Fig. 2). Using pharmacological inhibitors, we found that several protein kinases are necessary for the intracellular signal transmission after HIV exposure of HFA, but their order of activation requires further study (Figs. 3, 4, and 5). The activation by HIV requires, among others, the NF-κB and PTK signaling pathways; however, surprisingly, the C3 core promoter lacks NF-κB and SP binding sites. The requirement for functional NF-κB for the HIV effect on C3 was confirmed by overexpression of the NF-κB inhibitor I-κBα in astrocytes; adenovirus-mediated transduction of I-κBα into HFA, but not of control adenovirus, blocked nuclear translocation of NF-κB and prevented induction of the C3 promoter and mRNA. However, activation of the C3 promoter by HIV was absolutely dependent on the presence of the IL-6/IL-1β responsive element at −109 to −90 and its sub-domain −106 to −100. The neutralization of IL-6 by anti-IL-6 antibody abolished C3 promoter induction revealing an essential role for IL-6 in increased C3 protein synthesis in HFA upon HIV exposure. Earlier studies using astrocytic cell lines implicated cyclic AMP, protein kinase C, the IL-6/IL-1β responsive element in the C3 promoter, and transcription factor C/EBPδ, but not NF-κB or IL-6, in induction of C3 in transformed astrocytes by HIV [24, 26]. Our results demonstrate a novel auto-stimulatory pathway in the scheme of complement activation by HIV in primary astrocytes by indicating that HIV activates C3 in these cells indirectly through NF-κB-dependent induction of IL-6, which in turn acts as a second messenger in this pathway through induction of C3 transcription via the −109 to −90 IL-6/IL-1β responsive element in the C3 promoter.

Our findings from in vitro studies of HIV activation of C3 expression by HFA through IL-6 gain biological significance from observations that IL-6 is overexpressed in the brains of patients with HAND and the requirement for IL-1β/IL-6 element in the C3 promoter for its activation by HIV [26]. Independent studies of IL-6 activity show that when mice are genetically engineered to express IL-6 restricted to astrocytes through the GFAP promoter, C3 is upregulated in the brain but not in the peripheral tissues [67]. Astrocytes are now recognized as one element of the innate immune system, responding to brain injury not only by cytoskeletal changes to restrict neuronal injury but also by synthesis of effector proteins including cytokines, interferon-related proteins, and complement components [30]. Furthermore, astrocytes are increasingly recognized as an important HIV reservoir in patients at the presymptomatic stage of infection [66] and in SIV-infected macaques throughout the course of SIV disease [68]. What has proven particularly striking are findings in the last decade from multiple systems that decoration of neurons with complement components is a pivotal feature of synaptic pruning by microglia that occurs during normal development and that can underlie functional changes observed during distinct neuropathological disorders (reviewed in [31, 69]). C1q and C3 knockout mice exhibit impaired synapse elimination during brain development [41]; indeed without C1q, mice are susceptible to both spontaneous and induced epileptic seizures associated with excess excitatory synapses [70]. Synaptic loss in a mouse model of Alzheimer’s disease has been linked to aberrant expression of C1q which was further shown essential for soluble β amyloid synaptic toxicity [47]; in mice, West Nile virus infection of neurons drives their expression of complement components leading to pruning of pre-synaptic termini, a disease state absent in C1q or C3 knockout mice [50], and remarkably, C3 knockout mice failed to display even synaptic loss leading to neuronal loss and defects in memory observed during normal aging [71]. These observations suggest that overexpression of complement proteins in the brain, independent of other known neuropathogenic mediators such as amyloid plaques in AD or HIV Tat protein in HAD, may directly contribute to neuronal injury in diseased brain. Studies in the present work, along with previous reports [24–26], suggest that astrocytes may serve as an important source of neuropathogenic complement components in HIV-infected brain. Our discovery that IL-6 expression is central to C3 induction by HIV in astrocytes (Figs. 7 and 8) may provide an avenue to new therapeutic investigations in this common pathway to diverse brain diseases plaguing mankind [72, 73].

## Conclusions

The study presented here indicates that HIV induces C3 expression in primary human astrocytes indirectly, through NF-κB dependent induction of IL-6, which in turn activates the C3 promoter. HIV induction of C3 and IL-6 in astrocytes may contribute to HIV-mediated inflammation in the brain and cognitive dysfunction.

## Additional file

Additional file 1: Table S1. Supplementary Statistical Results. Result tables of statistical results for one-way ANOVA and Dunnett’s post hoc analysis. Each sheet in the Excel file contains the corresponding figure analyzed by this approach. (XLS 59 kb)
